# Executive Functions and Long-Term Metabolic Control in Adults with Phenylketonuria (PKU)

**DOI:** 10.3390/metabo15030197

**Published:** 2025-03-12

**Authors:** Anne Tomm, Alena G. Thiele, Carmen Rohde, Haiko Schlögl, Wieland Kiess, Skadi Beblo

**Affiliations:** 1 Center for Pediatric Research Leipzig, Department of Women and Child Health, Hospital for Children and Adolescents, University Hospital Leipzig, 04103 Leipzig, Germany; alena.thiele@medizin.uni-leipzig.de (A.G.T.); carmen.rohde@medizin.uni-leipzig.de (C.R.); wieland.kiess@medizin.uni-leipzig.de (W.K.); skadi.beblo@medizin.uni-leipzig.de (S.B.); 2 Department of Internal Medicine, Section of Endocrinology, Nephrology, Rheumatology, University Hospital Leipzig, 04103 Leipzig, Germany; haiko.schloegl@medizin.uni-leipzig.de; 3 Leipzig University Center for Rare Diseases, 04103 Leipzig, Germany

**Keywords:** phenylketonuria, executive functions, adults, metabolic control, dietary treatment

## Abstract

**Background/Objectives:** Phenylketonuria (PKU) is a rare inherited metabolic disorder caused by phenylalanine hydroxylase deficiency, resulting in highly elevated blood phenylalanine (Phe) concentrations, leading to neurotoxic effects. Despite advancements in treatment, adult patients with PKU may experience impairments in executive functions (EFs). This study investigates the influence of metabolic control across different life stages on EFs and sociodemographic outcomes in adult PKU. **Methods:** We conducted a monocentric study with 36 early-diagnosed and treated PKU patients (mean age: 34.8 years). EFs were assessed using the Test Battery for Attentional Performance (TAP) and the Tower of London (TL-D). Metabolic data were extracted from medical records, focusing on childhood and adulthood metabolic control, including Phe fluctuations. Sociodemographic data were collected via questionnaires. Statistical analyses explored relationships between EFs, metabolic control, and sociodemographic data. **Results:** EFs in the cohort were within the lower average range. Significant negative correlations could be observed between EF performance and dried blood Phe concentrations during childhood (ages 0–10 years) as well as current Phe concentrations and Phe variation. Elevated childhood Phe concentrations were associated with lower educational attainment. Sociodemographic characteristics, such as employment status and living arrangements, aligned with those of the general population. **Conclusions:** Optimal cognitive development in PKU requires good metabolic control, particularly in early childhood. In adulthood, while dietary restrictions may be relaxed, maintaining low and stable Phe concentrations is crucial for EFs. Consistent monitoring and tailored therapeutic approaches throughout life seem essential for optimizing metabolic and neurocognitive outcome in PKU.

## 1. Introduction

Phenylketonuria (PKU; ORPHA716) is classified as a rare metabolic disease, affecting the amino acid metabolism. A deficiency of the enzyme phenylalanine hydroxylase (PAH), which is responsible for facilitating the conversion of the essential amino acid phenylalanine (Phe) into tyrosine, is causative of this autosomal recessive disorder. Consequently, excess Phe accumulates in the bloodstream, leading to toxic effects on the central nervous system, compounded by a deficiency in tyrosine and the toxic accumulation of phenylketones. If left untreated, PKU causes intellectual disability, along with epileptic seizures and behavioral disturbances characterized by aggression or hyperactivity. Additionally, individuals with untreated PKU often exhibit eczema and a pale complexion, with light skin and hair [1,2,3]. Other causes of hyperphenylalaninemia related to cofactor metabolism are not part of the study population.

With the introduction of newborn screening in the late 1960s, enabling early diagnosis, a Phe-restricted diet can ideally be implemented within the first few weeks of life [4]. This protein-restricted diet must be maintained beyond childhood and, additionally, a supplementation of amino acid mixtures is necessary. Accordingly, the current target Phe levels for children (up to age 12) are 120–360 μmol/L, and for individuals older than 12 years, 120–600 μmol/L. This enables the attainment of almost normal motor and cognitive development [5].

Moreover, newer therapeutic approaches, including the administration of the cofactor tetrahydrobiopterin (BH4), administered as sapropterin hydrochloride, the injection of the enzyme supplementation therapy pegvaliase, or potentially in the future through sepiapterin (currently at primary endpoint in a pivotal Phase 3 clinical trial), provide relief or even the possibility of discontinuing the dietary restrictions for responsive patients [6,7,8].

PKU thus represents a prime example of a condition for which the development of modern science has provided a treatment that improves the global outcome for patients. When evaluating the success of treatment, the performance in later life is, of course, of particular importance. However, studies involving older adults with PKU have only been conducted in recent years, and the available data remain sparse. Preliminary studies present conflicting findings and indicate the possibility of minor limitations which may still affect the daily living of these patients [9,10,11,12,13]. Several studies have consistently shown that working memory abilities and executive functions in patients with PKU are significantly poorer across studies compared to healthy controls [14,15,16]. In contrast, language abilities, such as verbal fluency, often appear to be well preserved [15,17,18]. These results further emphasize that PKU presents a distinct neurocognitive profile that requires separate consideration, extending beyond the mere assessment of IQ, which aggregates various facets of neurocognitive abilities.

Therefore, we examined a homogeneous group, including some of the oldest patients with PKU, who received early diagnosis and treatment from our research site. Newborn screening, as well as biochemical and genetic testing, was conducted in accordance with the applicable guidelines. In this study, particular emphasis was placed on investigating functions relevant for daily living in relation to cognitive performance, not general intelligence. For this purpose, we assessed certain executive functions (EFs) and sociodemographic data, examining how both current and childhood metabolic control may influence cognitive performance in middle age.

Thus, we aim to explore the significance of sustained good metabolic control across different stages of life in providing optimal care and guidance for adult patients with PKU.

## 2. Materials and Methods

We conducted a monocentric study in which we assessed EFs using various test batteries, collecting sociodemographic data via questionnaires and data on metabolic control from medical records. Participation was offered to all patients with PKU 18 years or older, who were early-diagnosed, treated, and regularly followed up in the outpatient clinic for inherited metabolic diseases at the University Hospital Leipzig, Germany.

The study received ethical approval from Leipzig University Medical faculty’s ethics committee (registration number: 273/21-ek; date of approval: 5 July 2021) and has been registered with the German Clinical Trial Register (DRKS00032654) at the International Clinical Trials Registry Platform. Informed consent was obtained from all participants. EF tests were conducted after each patient‘s regular appointment for metabolic consultation. The duration of the tests, carried out in a standardized setting, was approximately 45 min.

### 2.1. Executive Function Testing

#### 2.1.1. Test Battery for Attentional Performance (TAP)

The TAP is a computer-based instrument designed to assess specific attentional functions in participants aged 10 years and older [19]. It comprises simple reaction paradigms where subjects respond to non-verbal, distinguishable stimuli by pressing a designated button. Performance criteria include, depending on the test, response time, error rate, and omissions. The TAP is well validated and includes normative data for various age groups and subtests. We selected the following five subtests, which were adapted based on previous findings in the literature and their relevance to everyday life, for our study: working memory (assessed using the n-back task), flexibility (evaluated through the set shifting task), divided attention (measured by a task requiring simultaneous attention to multiple processes), impulse control (assessed using the Go/NoGo task, which measures the ability to withhold inappropriate responses), and incompatibility (evaluated by tasks assessing interference due to stimulus-response incompatibility).

#### 2.1.2. Tower of London (TL-D—Turm Von London—German Version)

The TL-D assesses complex planning processes that require the identification and evaluation of numerous potential action options to achieve a desired outcome [20]. The task involves moving differently colored balls along rods of various lengths from a starting position to a target position using the fewest possible moves, with only one ball allowed to be moved at a time. The number of moves is recorded as raw data and converted into percentile ranks. A normative dataset is available for the number of problems solved in the age group of 18 to 65 years.

### 2.2. Metabolic Data

Data on current and long-term therapy (including Phe-restricted diet, BH4, or pegvaliase supplementation) and biochemical parameters were gathered from electronic medical records. With the exception of five patients, we successfully integrated Phe concentrations from childhood and adolescence into our calculations. To assess long-term metabolic control, all available dried blood Phe concentrations until study enrolment were analyzed. This resulted in a median of 272 Phe values per patient. Until 2005, blood values were measured using fluorometry or ion exchange chromatography; afterwards, dried blood analysis was performed by liquid chromatography/tandem mass spectrometry, as previously described [21,22,23,24].

In our principal research approach, we analyzed two life stages separately: under 18 years of age (*Phe childhood*) and over 18 years of age (*Phe adulthood*). These variables are represented by the mean of medians of all Phe values per year of life for each patient (0–18 years and from 18 years onwards, respectively), in analogy to previous studies [25]. Additionally, we illustrated fluctuations through the variable *Phe variation* (again, for both childhood and adulthood separately). *Phe variation* is defined as the mean of the standard deviation of all Phe values per year of life for each patient.

Additionally, this approach was employed in presenting the Phe values from different stages of childhood and adolescence (Phe 0–6, 6–10, and 10–18 years, including variation). We selected these age intervals based on the treatment guidelines previously implemented, when the majority of the patients were still underage [9]. To account not only for the blood value on the day of testing (*Current Phe*) but also for the overall recent metabolic control, we determined the median (*Recent Phe*) and the standard deviation (*Recent Phe SD*) of all Phe values from the previous two years (max. 10 values). Additionally, we recorded the duration from birth to start of treatment (*Duration*) and the first measured Phe value (*Newborn screening*). For the latter, the measurement method is comparable, whether the value was obtained via mass spectrometry or fluorometry [21]. Therefore, no distinction will be made in what follows.

### 2.3. Sociodemographic Data

Sociodemographic data, as well as data on dietary management and anthropometric measurements, were collected via questionnaires. This included information on the type of therapy (diet, pegvaliase, and BH4), Body Mass Index (BMI), school-leaving certificate, employment status, form of relationship, living arrangement, and number of children. The reference population was primarily based on data from surveys conducted as part of the micro census of the Federal Statistical Office of Germany [26,27,28,29,30,31,32,33,34]. In addition, information on the type of housing and on early retirement are based on data from the Federal Agency for Civic Education (*Bundeszentrale für politische Bildung*) and the German pension insurance fund (*Deutsche Rentenversicherung*), respectively [35,36].

### 2.4. Statistical Analysis

Statistical analyses were performed using the statistical software R 22 (R Core Team, 2024), including the following packages: readxl [37], corrplot [38], psych [39], openxlsx [40], rcompanion [41], rstatix [42], and coin [43].

The distribution of patient characteristics and sociodemographic data was analyzed in comparison to the reference population using Chi-square goodness-of-fit tests. We compared the test results for EFs of the complete patient group with the normative sample of the corresponding test (shown in the respective manual) via a one-sided Wilcoxon rank-sum test. For the comparison between genders, the Mann–Whitney U test was employed. The sample was divided into three educational attainment groups (lower secondary school, intermediate secondary school, and higher secondary school), for comparison between groups by using a Kruskal–Wallis test. We calculated Spearman correlations between metabolic control at different stages of life and EFs. Significance was accepted for *p* < 0.05.

For explorative purposes and to enhance clarity, we summed all twelve correlation coefficients related to EFs for each life stage to provide a clearer understanding of which life periods have a particularly strong impact on EFs.

## 3. Results

### 3.1. Patient Characteristics

A total of 66 patients with PKU were invited to participate in the study. Of these, 21 either could not or did not wish to spend additional time after their regular consultation, particularly given their long travel distances. Another nine patients spontaneously canceled their appointment without providing a reason and did not schedule a new appointment after the recruitment period had ended. In total, 36 patients (20 female) agreed to participate. Five of these were BH4-responsive (and treated for at least 14 years), and four had been successfully treated with pegvaliase (for at least one year), thus not requiring strict dietary treatment. A total of 27 patients received amino acid mixtures as protein substitutes. None of the participating patients in our study could be classified as mild hyperphenylalaninemia (mHPA) (ORPHA79651), thus not requiring any treatment. The average age at participation was 34.8 (SD: 10.9, range 18–53) years (see Table 1).

Anthropometric data did not deviate significantly from the reference population (see Table 2). No significant gender differences were detected, regarding assessments of metabolic control and sociodemographic data. Likewise, no differences in anthropometric data occurred when comparing different treatment groups (diet vs. BH4 or pegvaliase).

In terms of sociodemographic data, our sample differed from the normative comparison group. On average, the investigated patients with PKU tended to have higher educational attainment and fewer children compared to a normative sample. However, the frequency of full-time employment, as well as the distribution with regard to living arrangements, was comparable. It is also worth noting that all of our patients have obtained a school-leaving certificate. Additionally, our sample was more frequently in a relationship or married than the reference population (see Table 3).

### 3.2. Metabolic Control

Regarding metabolic control, median dried blood Phe concentrations during childhood were found to be in accordance with the recommended guidelines. However, Phe concentrations in adulthood, particularly the current Phe level, exceeded the recommended threshold (see Table 4).

*Age* was positively correlated with *mean Phe childhood* (*r =* 0.66, *p <* 0.05), indicating that the older the patient was at the time of testing, the poorer their metabolic control during childhood tended to be. In contrast, no correlation could be found regarding *age* in relation to *mean Phe adulthood* (*r =* −0.15, *p =* 0.38), *recent median* (*r =* −0.00, *p =* 0.99), or *current Phe* (*r =* −0.01, *p =* 0.95).

**Figure 1:**
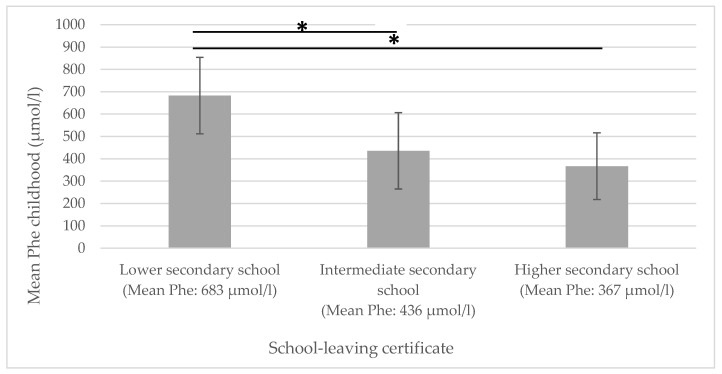
Mean Phe childhood in relation to educational attainment. Phe = phenylalanine concentrations in dried blood. * significantly different.

A significant negative correlation could be shown for *mean Phe childhood* and educational attainment: the higher the *mean Phe childhood* levels, the lower the educational attainment (see Figure 1).

**Figure 2:**
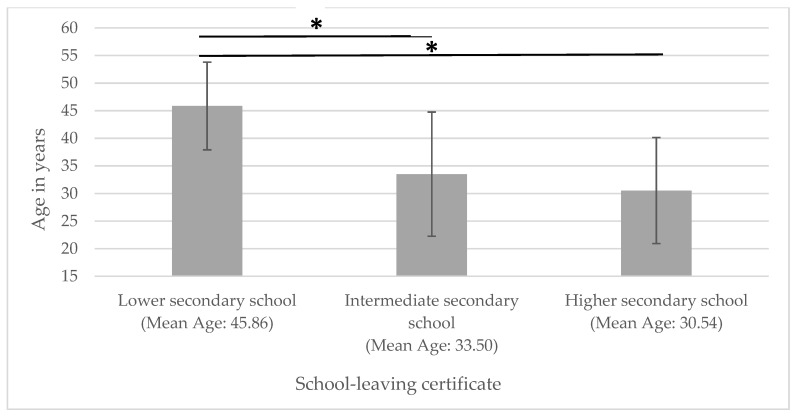
Age in relation to educational attainment. * significantly different.

Accordingly, patients with a lower secondary school diploma were significantly older than those with an intermediate secondary school or higher secondary qualifications (see Figure 2).

### 3.3. Executive Functions

**Figure 3:**
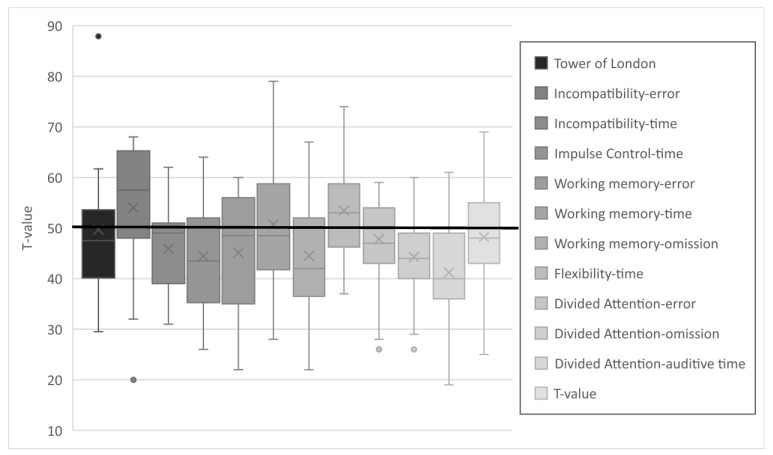
T-values of the subtests of the executive functions.

The results of the various subtests of the TAP and TL-D were in the lower average range (see Figure 3). Differences to the reference population were statistically significant in all but 3 of the 12 subtests: *working memory* (reaction time) and *divided attention* (errors and visual reaction time). The complete dataset is provided in the Supplementary Materials, Table S2. Due to the test design, it is not possible to compute a composite score for EFs. It appears that particularly the values for *auditory reaction time* are in the lower average range. This is in contrast to an average performance in *visual reaction time* and a near-average error rate. A similar trend was observed with respect to *incompatibility*, where patients made fewer errors but demonstrated reduced response times. An opposite trend was observed in *working memory*, where patients exhibited average speed but demonstrated a higher omission and error rate.

Gender differences were identified only in the subtest of divided attention (errors), where male patients made significantly fewer errors (median = 41 vs. 53), *z* (n1 = 20; n2 = 16) = −2.16; *p* = 0.03; *r* = 0.36).

### 3.4. Relation Between Executive Functions and Metabolic Control (Long Term/Concurrent)

Significant negative correlations were found between specific EFs and metabolic control at different life stages. Errors in the *working memory test* and the *TL-D*, as well as performance in tasks requiring *divided attention*, appeared particularly vulnerable to elevated Phe concentrations (the complete dataset is provided in the Supplementary Materials, Table S1. Most significant correlations found were of medium strength and ranged between −0.35 and −0.44. This applies, among others, to the *Phe levels at ages 0–6 years* and the *TL-D*, as well as *errors in working memory* (−0.44 and −0.41, respectively). Regarding the correlation between *Phe variation at age 6–10 years* and *working memory errors*, as well as between *recent Phe SD* and the *omissions at the divided attention test*, a strong significant relationship was observed (−0.51 for each).

In line with the average EF values reported above, no consistent correlations with Phe measures in the different life stages were observed regarding *(visual) reaction time* of *divided attention* and *working memory*. For the former, both positive and negative significant correlations were identified, while for the latter, no significant associations were found.

**Figure 4:**
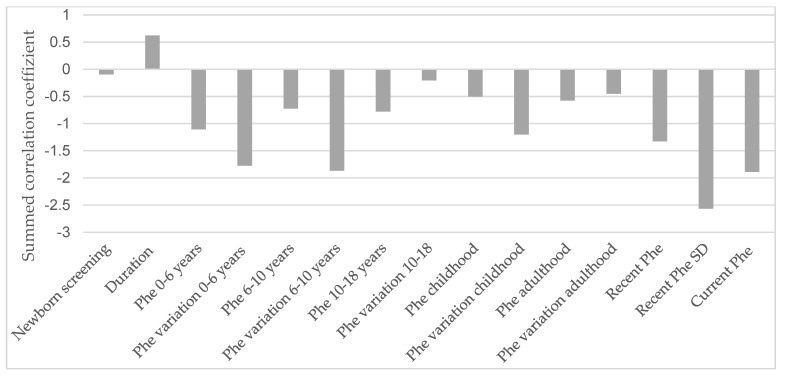
Influence of metabolic control in each life stage on the correlation sum score of the executive functions. Phe = phenylalanine concentrations in dried blood; SD = standard deviation. The underlying dataset is available in the Supplementary Materials (Table S1).

Figure 4 illustrates the influence of various life stages related to metabolic control on all described EFs in an explorative manner.

It is apparent that the current Phe level, recent SD, and Phe concentrations during the first six years of life, as well as variations in Phe levels between the ages of 0 and 10, have the most pronounced impact on current EF performance.

## 4. Discussion

Advancements in management have led to significant improvements in long-term outcomes for individuals with PKU. However, evidence indicates a close relation to an individual’s metabolic control. Numerous studies have investigated this subject, yielding heterogeneous findings: while some identify impairments in EFs compared to the general population, there remains considerable debate regarding the timing and mechanisms by which Phe concentrations exert their impact. Factors such as Phe concentrations at the time of assessment, long-term metabolic control, and fluctuations in Phe levels are topics of ongoing discussion [9,10,11,12,13].

Given the pivotal role of EFs in daily life, these findings are particularly relevant to understanding the long-term impact of PKU. As EFs facilitate the practical application of intellectual abilities, they should be given due consideration. Furthermore, it is crucial to consider sociodemographic factors, as Phe concentration can influence elements such as education and socioeconomic background and play a significant role in determining the success of therapy. Therefore, we evaluated EFs and sociodemographic factors in a cohort of adult patients with PKU treated in Leipzig, examining the influence of short- and long-term metabolic parameters on these factors. In our study, we found further confirmation of the importance of maintaining low Phe concentrations throughout childhood and demonstrated the correlation with educational success. Furthermore, we found that tasks related to complex planning or working memory are particularly affected as they were completed with either reduced time or increased error rates. This is consistent with findings from several other research groups [12,13,14,44].

In the consideration of potential negative influencing factors on the reduced EFs, elevated current Phe levels appear to be a significant factor. Fluctuations (expressed as variations) in Phe levels, particularly of recent levels, had a negative impact on EFs in our cohort. This confirms findings from other samples [14,25,45,46]. Furthermore, we found that high Phe concentrations at a younger age specifically influenced current performance. Regarding this aspect, the existing data are contradictory. Some study groups have observed a similar relationship: Feldmann et al. [11] showed that cognitive performance correlated significantly with the blood Phe concentrations during childhood and adolescence. Additionally, a meta-analysis by Fonnesbeck et al. [47] demonstrated that Phe levels in the age range of 0–6 years were particularly influential on current cognitive performance. Elevated Phe values beyond this period also had a negative impact on cognitive performance, though the effect was less pronounced. Our data thus constitute a synthesis of the findings from these studies. However, other studies found less influence of childhood blood Phe concentrations. In contrast, Phe levels during adolescence (12–17 years) and adulthood (18+ years) were associated with current cognitive task performance [18,48].

Examining this interdependence in adults is particularly relevant, as it is well established that both the density and volume of white matter, and consequently the capacity for EFs, naturally decline in middle adulthood [49,50]. Considering the inherent cognitive decline in later life, this further underscores the critical importance of monitoring PKU patients in later stages of life, as initial studies provide indications of age-related cognitive deterioration [51]. Optimal metabolic control during adulthood may offer an opportunity to prevent premature dementia. Furthermore, it must, of course, be considered that socioeconomic factors can influence Phe levels at any age. For instance, a negative correlation has been observed between current Phe values in children and parental education, which seems understandable given the complexity of therapy for this condition [52,53]. When the individual treatment goals are determined, this consideration should be actively addressed and integrated into the care of adult patients.

The focus of this study was to investigate the influence of metabolic control during different stages of life on EFs in adulthood. While inclusion was offered to all early treated individuals with PKU, one has to assume that only those interested in good care could be studied. In addition, those with loss of follow-up over time could not be reached. This bias may have influenced our results. While introducing a control group of matched peers would have been valuable, we chose a comparison to the general reference population, which serves as the intended standard in this context. For the tests used, data from large normative samples are available, which cannot be matched numerically by a control group recruited here. Therefore, we consider the data to be highly representative. Additionally, it was not possible to include patients with mild hyperphenylalaninemia (mHPA) in the study, as they are typically not undergoing treatment and therefore often not followed up in an outpatient clinic on a regular basis.

Moreover, to better align the assessed functions with practical, everyday applications, future research should investigate fine motor abilities, as deviations in this domain have been documented in prior studies [5,10,13]. This was not feasible in our study due to the extensive number of tests and questionnaires that were administered.

Sociodemographic data, however, seem to increasingly align with those of the general population. Educational attainment and employment status appear to be even above average. Furthermore, a higher proportion of individuals were in a relationship or married compared to the reference population. This is likely due to the young age of our sample, where divorce and widowhood rates are low, and the number of students or apprentices is relatively high. The living arrangements seem comparable. It should be noted, however, that of the six individuals in our sample who lived with their parents, five were under 24 years old. This further underscores the improved independence currently observed in older PKU patients. Consequently, unlike previous research that found significant differences in relationship status, independent living arrangements, and academic achievement, our results indicate a positive trend within our cohort of patients with PKU [54,55]. These findings are consistent with recent studies that show improvements in educational attainment, income, and independence among individuals with PKU [56,57,58]. Nonetheless, the number of children is considerably below the population average, consistent with Klimek et al. (2020) [57]. Again, it is important to consider the relatively young average age of our sample.

We observed a significantly poorer level of educational attainment with increasing age in our cohort. This is attributable, alongside the significantly lower Phe concentrations in the younger cohort, to markedly improved therapy conditions and the continuous advancement and diversification of dietary supplements over the past two decades [59]. Additionally, it should be noted that recent decades have seen a general increase in the prevalence of higher education degrees within the overall population [26].

In relation to anthropometric data, our tested sample did not reveal any significant deviations from the normative population. In addition, no influence of the treatment strategy on anthropometric data could be identified. However, women in our sample showed a tendency to be more likely overweight compared to men. This observation is consistent with earlier research that also found no significant differences between patients with PKU and the reference group, but noted a similar trend concerning women with PKU [60,61,62]. Positive influencing factors in this context include maintaining low Phe concentrations and the intake of amino acid mixtures, both of which are associated with a lower body weight [63,64]. Men in our sample made fewer errors than women in the divided attention subtest of the TAP. This difference was not expected given the normative data of the test battery and study population. However, studies investigating multitasking have shown that men and women employ different strategies when approaching such tasks [65,66,67]. Metabolic control, however, does not appear to be the underlying cause, as it does not differ between the sexes in any life stage.

Taken together, it is of the utmost importance to ensure stable and continuous care for patients with PKU into adulthood, while emphasizing the significance of maintaining good metabolic control. Due to the possibility that patients with PKU do not always perceive fluctuations in their Phe levels, regular monitoring is essential [68]. Moreover, it emphasizes the need for tailored therapeutic approaches to optimize and balance both metabolic and neurocognitive outcomes. Further research is necessary to explore the mechanisms underlying the observed EF variability and to assess long-term effects of emerging treatments.

## 5. Conclusions

It appears that the foundation for cognitive development is established predominantly during childhood (up to 10 years of age), underscoring the critical importance of maintaining good metabolic control during this period. In adulthood, while dietary restrictions may be relaxed, it remains essential both to avoid general extensive fluctuations in Phe concentrations and to prevent the deterioration of metabolic control, as these directly affect performance in specific executive functions on an everyday basis. Further research in this field in an older PKU population should be performed when these patients have reached advanced age to elucidate possible long-term consequences.

## Figures and Tables

**Table 1:** Characterization of the population.

n		Total (36)	Female (20)	Male (16)
Mean age in years (SD)		34.8 (10.9)	37.4 (11.0)	31.6 (9.8)
Age at diagnosis in days (SD)		17.9 (11.5)	19.3 (14.7)	16.4 (8.9)
Amino acid mixture supply		27	14	13
Therapy				
	Diet alone	27	15	12
	BH4	5	3	2
	Pegvaliase	4	2	2

Available data for *Age at diagnosis*: female = 16; male = 14 patients.

**Table 2:** Anthropometric description of the sample at the time of investigation.

	Mean (SD)					
	All Patients	Reference Group ^a^	Female Patients	Reference Group ^a^	Male Patients	Reference Group ^a^
BMI (kg/m^2^)	26.9 (6.4)	26.0	28.8 (6.8)	25.2	24.6 (4.9)	26.8
Height (cm)	170.0 (9.6)	172.5	164.7 (6.8)	165.8	176.7 (8.2)	178.9
Weight (kg)	77.8 (19.2)	77.7	78.1 (19.5)	69.2	77.4 (18.8)	85.8

^a^ = reference groups according to [34]. BMI = Body Mass Index; SD = standard deviation.

**Table 3:** Selected sociodemographic data.

Sociodemographic Data (n)		Study Participants in % (n)	Mean Age per Subgroup (in Years)	Reference Population in %	Chi-Square Test Statistic	*p*-Value
School-leaving certificate (36) ^a^					11.99	0.007 *
	None	0 (0)	-	5.3		
	Lower secondary school	19.4 (7)	45.9	24.4		
	Intermediate secondary school	44.4 (16)	33.5	31.4		
	Higher secondary school	36.1 (13)	30.5	40.0		
Employment (36) ^b^					21.819	<0.001 *
	Unemployed	2.78 (1)	38.0	5.46		
	Early retirement	2.78 (1)	48.0	3.52		
	Student	11.11 (4)	26.0	6.11		
	Trainee	8.33 (3)	19.7	2.54		
	Part-time employment	16.67 (6)	35.5	25.44		
	Full-time employment	58.33 (21)	37.7	56.93		
Form of relationship (33) ^c^					10.455	<0.005 *
	Single	30.30 (10)	29.6	31.79		
	In a relationship or married	63.64 (21)	37.3	50.98		
	Other (widowed/divorced)	6.06 (2)	45.5	17.23		
Living arrangement (35) ^d^					1.5075	0.6805
	Alone	17.14 (6)	34.0	20.80		
	Shared living	5.71 (2)	30.0	5.00		
	With family/partner	60.00 (21)	38.3	60.70		
	With parents	17.14 (6)	24.0	13.50		
Mean number of children (35) ^e^		0.46		1.46		
Number of children ^f^					50.431	<0.001 *
	0	65.71 (23)	30.6	33		
	1	22.86 (8)	39.6	35		
	2	11.11 (4)	48.0	21		
	3+	0	-	11		

Reference groups according to a = [26]; b= [27,28,29,30,36]; c = [31]; d = [35]; e = [33]; f = [32]. * significantly different.

**Table 4:** Metabolic control.

	No. of Patients:	No. of Measurements per Patient: Median (min–max)	Median Phe Concentration in µmol/L (IQR)
Newborn screening	31	1	1755 (1074)
Phe 0–6 years	32	6 (3–6)	332 (217)
Phe 6–10 years	32	4 (2–4)	321 (436)
Phe 10–18 years	31	8 (3–8)	546 (414)
Phe childhood	32	18 (9–18)	431 (308)
Phe adulthood	36	12 (2–31)	725 (214)
Phe variation 0–6 years	32	6 (3–6)	179 (72)
Phe variation 6–10 years	32	4 (2–4)	161 (67)
Phe variation 10–18 years	30	8 (3–8)	104 (50)
Phe variation childhood	32	18 (8–18)	154 (62)
Phe variation adulthood	36	11 (1–31)	112 (57)
Recent median	36	8.5 (3–10)	768 (442)
Recent SD	36	8.5 (3–10)	163 (82)
Current Phe	36	1	845 (646)

IQR = interquartile range; min = minimum; max = maximum; Phe = phenylalanine concentrations in dried blood; SD = standard deviation.

## Data Availability

The data presented in this study are available on request from the corresponding author.

## Supplementary Materials

The following supporting information can be downloaded at: https://www.mdpi.com/article/10.3390/metabo15030197/s1, Table S1: Spearman correlation of metabolic control and executive functions, Table S2: Comparison of patients’ results with the reference median of 50 (ToL and TAP).
