# Supplementary material for: Executive Functions and Long-Term Metabolic Control in Adults with Phenylketonuria (PKU)
Source: Metabolites. 2025 Mar 12;15(3):197. doi: 10.3390/metabo15030197 (PMC11943845; doi:10.3390/metabo15030197)
Supplement: Supplementary file 1 [file metabolites-15-00197-s001.zip › metabolites-3468754-supplementary.pdf]

Supplements (Tables S1 and S2)

Table S1. *Spearman correlation of metabolic control and executive functions*

	Percen-tile ToL	Incompatibility		Impulse control Task	Working memory			Flexi-bility	Divided attention			
		Errors[b]	Reaction time[b]	Reaction time[d]	Errors[d]	Reaction time[d]	Omissions[d]	Reaction time[c]	Errors[d]	Omissions[d]	(Audit.) reaction time[b]	(Visual) reaction time[b]
**Newborn screening**	0.080	-0.026	-0.303	-0.003	-0.243	0.246	-0.045	0.196	-0.092	-0.099	0.106	0.089
**Duration**	-0.044	0.052	0.195	0.065	-0.304	0.214	0.256	0.205	-0.290	-0.051	0.056	0.267
**Phe 0-6 years**	**-0.442***	-0.250	-0.087	0.053	**-0.406***	0.270	0.128	0.079	-0.233	-0.316	-0.072	0.172
**Phe 6-10 years**	-0.285	-0.194	0.042	0.080	**-0.420***	0.222	-0.098	-0.007	0.006	-0.352	-0.125	**0.410***
**Phe 10-18 years**	-0.084	-0.225	-0.028	-0.012	-0.140	0.107	-0.037	0.006	-0.195	-0.239	-0.186	0.256
**Phe childhood**	-0.258	-0.220	0.025	-0.001	-0.288	0.268	0.036	0.027	-0.087	-0.240	-0.152	**0.390***
**Phe adulthood**	0.055	-0.052	-0.065	-0.238	0.052	-0.116	-0.132	-0.016	0.018	0.046	-0.088	-0.039
**Phe variation 0-6 years**	-0.220	-0.172	-0.122	-0.117	**-0.387***	-0.138	-0.150	-0.199	-0.256	-0.132	-0.034	0.151
**Phe variation 6-10 years**	-0.126	-0.196	-0.070	-0.144	**-0.508***	-0.119	-0.326	-0.109	-0.244	-0.347	0.040	0.281
**Phe variation 10-18**	0.181	-0.254	0.069	-0.074	-0.312	0.122	-0.031	0.009	-0.259	-0.012	-0.053	**0.410***
**Phe variation childhood**	-0.109	-0.215	-0.016	-0.090	**-0.418***	0.038	-0.131	-0.093	-0.284	-0.217	-0.059	**0.392***
**Phe variation adulthood**	-0.091	-0.163	0.041	0.022	-0.214	0.020	-0.055	0.044	-0.216	-0.077	0.010	0.230
**Recent Phe**	0.082	-0.224	0.107	-0.200	-0.140	-0.114	-0.203	-0.214	-0.045	0.024	**-0.346***	-0.052
**Recent Phe SD**	-0.221	**-0.401***	-0.241	-0.107	-0.183	-0.218	0.081	-0.022	-0.146	**-0.509***	-0.252	**-0.345***
**Current Phe**	-0.044	0.052	0.195	-0.183	-0.299	-0.269	-0.317	-0.272	-0.089	-0.139	**-0.422***	-0.102

Note. * $p < .05$.

Supplements (Tables S1 and S2)

Table S2. *Comparison of patients' results with the reference median of 50 (ToL & TAP)*

		Observed median	Z-value	Effect size	P
**Main test**					
	Subscore				
**Percentile ToL**		47.5	-2.56	0.43	.01*
**Incompatibility**					
	Errors	57.5	-2.10	0.36	.04*
	Reaction time	49.0	-2.44	0.43	.01*
**Impulse control Task**					
	Reaction time	43.5	-2.82	0.49	.005*
**Working memory**					
	Errors	48.5	-2.28	0.41	.02*
	Reaction time	48.5	-0.06	0.01	.94
	Omissions	42.0	-2.40	0.40	.02*
**Flexibility**					
	Reaction time	53.0	-1.97	0.34	.05*
**Divided attention**					
	Errors	47.0	-0.89	0.15	.37
	Omissions	44.0	-4.03	0.73	<.001*
	(Audit.) reaction time	40.0	-4.00	0.70	<.001*
	(Visual) reaction time	48.0	-1.14	0.20	.26

The calculation was based on a one-sided Wilcoxon rank-sum test; * $p < .05$.
